# Towards “Groundtextual” Public Health: The Need for a Critical and Transformative Approach

**DOI:** 10.3389/phrs.2021.1604639

**Published:** 2021-12-31

**Authors:** Kesavan Rajasekharan Nayar

**Affiliations:** Global Institute of Public Health (GIPH), Thiruvananthapuram, India

**Keywords:** groundtextual public health, theory, pedagogy, philosophy, public health challenges

## Introduction

Public health theory, practice, and education that do not relate to the context become irrelevant. “Groundtextual” is a neologism of grounded and contextual public health which has both universalistic and particularistic elements in its package and should be capable of responding to local challenges. The purpose of this paper is to reflect on the current state of public health as it is a popular course in many universities and institutes. In any context, public health with a universalistic framework, strategy, or theory and methods needs to face the challenge of the diversities across regions and states. Experience in research with such diversities also prompts this paper as data from such different contexts make it a challenging task to develop uniform strategies. It is also extremely challenging because, in many instances, lack of data reduces public health to a “silent science.” Public health is now reduced to a data science. Especially, the prominence given to statistics, quantitative applications, and some broad theoretical notions means that scholars are unable to respond given the absence of reliable data on many infections except some easy-to-implement microanalyses. However, such prominence of quantification gives it an appearance of a universal science.

There exists an assumption among public health scholars, termed as a “laminar view,” which assumes that if one adopts a mechanical methodological approach similar to some of the normal sciences, it is possible to capture the phenomena consisting of issues related to health and health services in an uninterrupted flow. However, this is a myth as there are many conflicting spheres in public health especially when many social, cultural, and political contexts influence the phenomena. Most of the decision-making within the health services system is taken within the political sphere and is largely eminence-based because of the priorities and imperatives of the sphere itself. Such conflicts between the spheres and levels may not be revealed or may be captured only superficially in so-called mythicized and pedantic surveys and statistical research. The social sphere is also difficult to capture unless the researchers have unusually creative as well as responsive abilities. It is unrealistic to assume that any single study should or can investigate the entire gamut of the process and dynamics of the public health phenomena especially because of its complexity. The need for an *iterative* process which moves from simple level exploratory approaches to more complex and abstract constructions is to be recognized. But this is the missing link that gets lost in the present race for outputs which can be categorized as market-decided research, the so-called flourishing “knowledge market.”

This commentary is intended to reflect on these challenges and to create reflexive responses by the scholars in public health. This is especially important given the observation regarding the “silence of majority” of public health scholars to effectively intervene in or being cavalier of issues, both conceptually and practically in responding to many public health issues in the world including the pandemic, except for some mechanically executed research. Such silence has led to the dominance of self-appointed experts and/or eminence-based public health. This is indeed a complex issue as exposing the weaknesses with some clarity requires consistent dialogue, but let me begin my initial reflections by stating the many paradoxes in public health.

## Philosophical Paradoxes

Public health has not gone beyond “the right to health” and ethics in its philosophical outlook. The current crisis has revealed and exposed many weaknesses of this outlook, notwithstanding its positivity. This outlook always emphasized the need for provision of care as the foremost issue which resulted in making services available and creating institutions. It also reaffirmed the statistical face of public health eclipsing other key determinants of the scenario. This reaffirmation, for instance, as in the case of the present pandemic, has been partial as it could only access some generally agreeable data while hiding the more important social determinants as well as data which could lead to political discomfort.

Evidently, public health philosophy is teleological and outcome-determined [1]. Therefore, it has a larger practical outlook because the discipline is assessed on the basis of what is achieved and in terms of the benefits that are accrued due to the actions. But over time, we see the development of a dominant magic bullet and vertical approach to health. However, politics play an important role in public health discourse and actions. The efforts to make it scientifically neutral only make it irrelevant. Most research papers published in journals end up ritualistically stating practical and policy implications of their data, and they are also assessed largely in terms of this rather than how they add up to the public health knowledge storehouse.

The philosophy has undergone some changes due to shifts in the larger macroeconomic scenarios in the world and the new ideological elements entering the public health discourse. But it has also brought the “radical skeptic” to the forefront who was not averse to data but skeptical of the extreme empiricism and positivism which engulfed public health and the so-called “ritualistic and set” patterns of data gathering. Such scholars, based on their critical wisdom, examined new initiatives. They tried to change the earlier approaches of health services which proclaimed them as a right, including statements like “health services to be provided at the doorstep of the people irrespective of paying capacity,” etc. However, already critically analyzed shifts such as insurance or privatization were not explicit but hybrid so that some acceptability could be generated gradually. The biggest weakness of this new practical philosophical outlook of public health is the assumption of universality and its disconnect with people and the social structure. It is in this context that a critical orientation is necessary for conceptualizing transformative public health and a pedagogy appropriate for such public health.

## Critical Thinking and Qualitative-Qualitative Divide

It is easy to suggest the need for critical thinking in public health. However, it requires a clear road map and an in-depth understanding of ground level issues and their interrelationships. One of the issues which has almost taken over public health is the quantitative approach. However, the tension between quantitative and qualitative applications in public health is a perennial issue. The “number representations” become inadequate for public health while “exploring new ways in handling problems”; although there are possibilities of critical thinking even with a quantitative approach [2]. However, this is not realized by “quantitative disciplinarians.” The inadequacy is increasingly realized during the pandemic and it is time to update the existing repertoire of public health with an integrated holistic view which accepts a quantitative-qualitative synergy.

The absence of critical thinking also leads to inadequate problematization of issues despite some attempts at mechanically subscribing to a mixed method approach etc. while deciding the methodology. Many public health problems require careful problematization which means that health or ill-health have to be grounded and made relevant to the context. This has become increasingly applicable with respect to the present pandemic when the virus tends to act in different ways according to the context. Transformative public health and a pedagogy with such an orientation have to take into consideration such factors to make it relevant and useful.

## Pedagogical Paradoxes

If you state in epidemiological language, it becomes apparent that the relationships between humans and disease-causing agents in public health science are much more complex than the present understanding. This complexity needs to be addressed starting with the pedagogy which still depends on its ancestral fields such as Tropical Medicine and Hygiene, Preventive and Social Medicine, and their new avatar Community Medicine. This complexity cannot only be addressed by the methodological armor, which public health mechanically uses mostly drawn from these lineage disciplines and some liberally drawn from social sciences including qualitative methodology. The mechanical approach may be an under representation when you examine the so-called syllabi of public health. It actually reflects the lack of real interest and non-investment of any commitment. It is not my view that this complexity is not realized by those who develop public health syllabi in various institutions, but in many cases, it is preached without any sincerity or sensitivity. The paradox in pedagogy arises due to the divide between theory and practice as the teachers themselves are not aware of the key dimensions and theoretical concepts which are needed to understand “health in society.” Fundamentally, the dominance of technique-driven training is due to this limitation. There is also a trend of “bureaucratic pedagogy” where finishing portions, set patterns of interactions, and routinization of research are encouraged. Any interventions in the form of critical appraisals are looked down upon as serious violations within such a degraded academic culture.

A transformative approach cannot be addressed easily due to the limitations discussed in this commentary, but at least some steps could be taken to narrow the divide. It is now time to practice what has been preached regarding the complexity and generate a pedagogy which is “groundtextual” and which can respond to present-day public health challenges more effectively. Only then can public health overcome its silent status and take on the role of a transformative science.
